# Use of industrial liquid silicone: a scoping review

**DOI:** 10.1590/acb395624

**Published:** 2024-10-07

**Authors:** Ayla Gerk, Luiza Telles, Madeleine Carroll, Maria Eduarda de Freitas Mesquita do Nascimento, Rafaela Góes Bispo, Bruno Felipe Santos de Oliveira, Saulo Mendes, Sophie Nouveau Fonseca Guerreiro, Abbie Naus, Cristina Pires Camargo

**Affiliations:** 1 Harvard Medical School – Program in Global Surgery and Social Change – Boston (MA) – United States of America.; 2 McGill University – Faculty of Medicine and Health Sciences – Montreal (QC) – Canada.; 3 The Gender Equity Initiative in Global Surgery – Boston (MA) – United States of America.; 4 Instituto de Educação Médica – Rio de Janeiro (RJ) – Brazil.; 5 Universidade Estadual do Ceará – Mestrado Profissional em Ensino na Saúde – Fortaleza (CE) – Brazil.; 6 Universidade Federal da Bahia – Faculdade de Medicina – Salvador (BA) – Brazil.; 7 Universidade Federal da Paraíba – João Pessoa (PB) – Brazil.; 8 Comitê Técnico Estadual de Saúde LGBT do Rio Grande do Sul – Porto Alegre (RS) – Brazil.; 9 Universidade de São Paulo – Faculdade de Medicina – São Paulo (SP) – Brazil.

**Keywords:** Plastic Surgery Procedures, Silicone Oils, Silicones, Public Health, Surgery, Plastic

## Abstract

**Purpose:**

Illicit cosmetic injections remain highly prevalent and can cause serious complications, including death. We aimed to explore existing literature regarding the use of illicit cosmetic injections globally.

**Methods:**

We searched six databases with no language restriction from inception to 2022. We included all articles focused on adult patients of any gender who received any illicit cosmetic injection. Screening and data extraction followed standards from the Preferred Reporting Items for Systematic Reviews and Meta-Analysis Extension for Scoping Reviews guidelines.

**Results:**

After screening 629 abstracts and 193 full texts, 142 citations were included. We identified articles from 28 countries and three multi-country studies. Most were from high-income (75.3%) and upper-middle-income countries (21.8%). Of all patients whose gender identity was described, 49.9% were transgender women, and 40.8% were cisgender women. The anatomic regions most frequently injected were the buttocks (35%) and the breast (13.3%). The most frequently described complications were granuloma (41.5%), dermatological problems (41.5%), infection (35.9%), and pulmonary complications (34.5%).

**Conclusions:**

We observed the impact of illicit silicone injections, particularly on cisgender women and transgender individuals. Existing barriers must be addressed, including healthcare prejudice and inadequate knowledge about care for gender minorities. This will require educating at-risk groups and enhancing policies to regulate these procedures.

## Introduction

Throughout history, many societies have emphasized the value of physical beauty to the point that people are willing to risk their health to alter their appearance. This remains the case nowadays, with a myriad of illicit cosmetic procedures available to anyone willing to pay. One such procedure that has gained increasing popularity is the use of fillers with liquid injectable silicone (LIS)^1^. Plastic surgery associations have repeatedly discouraged the use of LIS for cosmetic improvement due to the significant associated health risks^2^.

In 2016, the American Society of Plastic Surgeons launched a public awareness campaign in response to the rise in deaths related to this illicit practice^2^. LIS often leads to local and systemic complications, ranging from non-esthetic outcomes, silicone granulomas, skin necrosis to severe infections, pulmonary embolism, and even death^2^^–^ 4.

Despite the risks associated with silicone injection, illicit and even some licit practitioners, both domestic and international, are willing to offer these services at a fraction of the cost of other cosmetic body contouring procedures^2^. The utilization of industrial liquid silicone to achieve aesthetic transformations in body contouring has persisted in underground circles for over six decades, and unfortunately, although banned for large-scale enhancement, the illicit use of fillers still thrives globally^2^^,^ 5.

Worldwide, the illicit use of fillers has been documented, particularly in Asian and South American countries, with higher rates in women and transgender individuals^4^^,^ 6 ^,^ 7. The illicit use of LIS has increased, not only in pursuit of aesthetic standards, but also in attempting to fulfill unmet gender affirmation needs1. For instance, a Brazilian study showed an illicit injection prevalence of 49% among transgender and *travesti* populations^8^. The medically underserved patient population appears particularly vulnerable to these clandestine practices^9^. Moreover, it has been documented that many people who experience complications from the procedure avoid seeking help in the health care system9. This might be related to prejudice in health care services and a lack of adequate health care system protocols and provider knowledge to attend to transgender and other vulnerable populations.

Although there is existing literature on the topic, a gap in knowledge in understanding the global landscape of this problem remains. Therefore, in this scoping review, we aimed to map the literature on complications related to the use of LIS and illicit filler substances worldwide and recognize the communities most impacted by these practices.

## Methods

### Study design and aims

This scoping review was reported according to the Preferred Reporting Items for Systematic Reviews and Meta-Analysis extension for Scoping Reviews (PRISMA-ScR)^10^. We primarily sought to investigate the complications provoked by silicone injections and illicit filler substances worldwide. Our secondary aim was to map the impact of this practice and its complications on gender minorities.

### Eligibility criteria

#### Participants

The inclusion criteria for the studies were participants older than 18 years old, regardless of sexuality and gender identity, who received an injection of any illicit filler substance (silicone, polymethacrylate, paraffin, etc.), by any provider (licensed or unlicensed), in any part of the body.

To be included, studies must deliver primary data with an outcome related to the use of industrial liquid silicone in this population. A comprehensive search strategy is outlined to identify studies on the prevalence of complications due to industrial silicone procedures in Brazil and worldwide.

The exclusion criteria were studies that included legal cosmetic procedures performed by licensed providers, those focused on implant-based cosmetic surgery, and studies in languages other than Portuguese, English, or Spanish. Regarding study design, we excluded commentaries, letters to the editor, viewpoints, editorials, op-eds, opinion papers, and non-human research.

#### Information sources and search strategy

Articles addressing our research question were identified by searching Medline/PubMed (National Library of Medicine, National Center for Biotechnology Information), PsycInfo, CINAHL, Web of Science (Clarivate), and Cochrane. Controlled vocabulary terms (e.g., Medical Subject Headings, Emtree, CINAHL Subject Headings, CABI Thesaurus) were included when available and appropriate. Due to the authors’ language proficiency, a language limit was applied to include English, Portuguese, and Spanish.

### Selection process

References were imported into Covidence online software, in which additional duplicates were removed11. In Covidence, two independent reviewers screened titles and abstracts. Full texts of potentially relevant sources were retrieved and imported into Covidence. Two independent reviewers screened the full text of selected citations according to our eligibility criteria. Reasons for excluding sources of evidence in full text that did not meet the inclusion criteria were recorded. Additional reviewers resolved disagreements at each stage of the selection process.

### Data extraction

Two independent reviewers extracted the data for each citation using Covidence11. A third reviewer resolved disagreements.

The following items were charted from each included source of evidence: title; year of publication; language in which the full text is available; country of the study population; study design; type of source; total number, age, gender, professional, location, illicit filler, volume injected, adverse events, and treatment used. Strategies, measures, and lessons for these illicit practices were also extracted and analyzed.

### Data synthesis

We used frequencies and percentages to describe the characteristics of the included studies. Quantitative and qualitative data from the studies were the primary data sources to respond to the review’s objectives.

## Results

**Figure 1:**
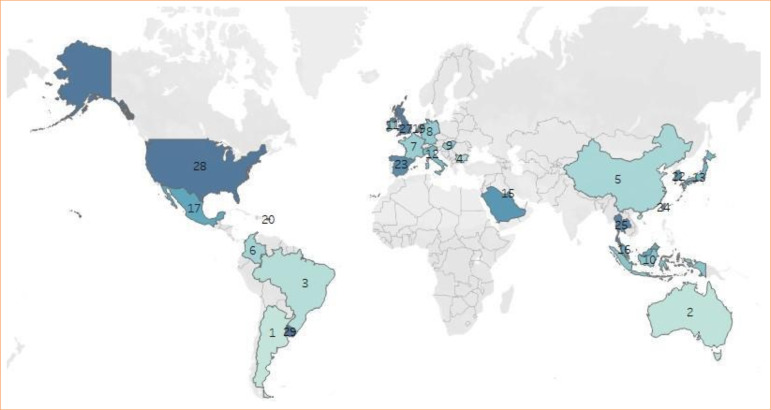
Geographic heat map of the country of origin of included studies. The countries are shaded according to the number of studies published.

Source: Elaborated by the authors.

The search yielded 638 results (Suppl. Mat. 1). Duplicates were identified and automatically excluded from the dataset. Nine duplicate entries were removed from further consideration. Two reviewers performed abstract/title screening using the Covidence platform. During this stage, 436 irrelevant results were excluded based on their abstracts and titles. We identified 142 articles that met inclusion criteria (Appendix 2). In Table 1, we detail the general characteristics of all studies. Most studies were case reports, 65% (92/142), and 23% (16/142) were case studies. Studies were from 28 different countries (Fig. 1), the vast majority of which were from high-income countries (107, 75.3%), and none were from low-income countries or lower-middle-income countries. The mean age of participants was 38 years old, and the number of participants varied between one and 234, with 88.7% of studies including only one participant (126/142). As for gender identity, 51% (72/142) included cisgender women, 18% (25/142) cisgender men, and 13% transgender women (18/142) (Table 2). Of all patients included whose gender identity was described, 49.9% were transgender women (520/1042), and 40.8% cisgender women (425/1042).

**Table 1:** Characteristics of the included studies.

Characteristic of studies	n = 142 (%)^1^
**Country income level**	
High-income countries	107 (75.3)
Upper-middle-income countries	29 (21.8)
Lower-middle-income countries	0 (0)
Low-income countries	0 (0)
Multiple countries of different income class included	3 (2.1)
**Geographic region**	
East Asia and Pacific	23 (16.2)
Europe and Central Asia	19 (13.4)
Latin America and Caribbean	24 (16.9)
Middle East and North Africa	2 (1.4)
North America	70 (49.3)
Multiple regions (North America, Latin America and Caribbean)	2 (1.4)
Multiple region	1 (0.7)
**Study design**	
Commentary	1 (0.7)
Letter to the editor	1 (0.7)
Narrative review	1 (0.7)
Case-control study	1 (0.7)
Case report	93 (65.5)
Case series	36 (23.4)
Cohort study	9 (6.3)
Cross-sectional study	2 (1.4)

*Median (interquartile range); n (%). Source: Elaborated by the authors.

Regarding providers responsible for the procedures (Table 2), 50% (71/142) of the articles mentioned they were unlicensed, and 40.1% (57/142) of studies did not mention the provider’s background. In 10.6% (15/142) of the studies, procedures were reported to occur in medical facilities.

**Table 2:** Study population characteristics.

Population characteristic	n = 142 (%)*
**Mean age of the participants**	38 (29.45)
**Gender identity of the participants**	
Cisgender woman	72 (51)
Cisgender man	25 (18)
Multiple gender identities	17 (12)
Not specified in the study	5 (3.5)
Transgender man	5 (3.5)
**Transgender woman**	
Substance injected	18 (13)
Acrylate	1 (0.7)
Biopolymer	1 (0.7)
Castor oil	1 (0.7)
Cod liver oil	1 (0.7)
Liquid silicone	106 (75)
Methacrylate	1 (0.7)
Mineral oil	1 (0.7)
Multiple substances injected	11 (7.7)
Not specified	10 (7)
Polyacrylamide	4 (2.8)
Polysiloxane	3 (2.1)
Vitamins A/E	2 (1.4)
Who performed the illicit procedure	
Not described	57 (40)
Physician	4 (1.4)
Self-administered	9 (6.3)
Surgeon	1 (0.7)
Unlicensed professionals	71 (50)

* Median (interquartile range);

n (%). Source: Elaborated by the authors.

As for the anatomical injection sites, 30 studies (30/142, 21.1%) reported injections in multiple locations in the same patient. Body locations most commonly described were the breasts (19/142, 13.3%), the buttocks (49/142, 34.5%), the face (20/142, 14.0%), the thighs (13/142, 9.1%), and the penis/scrotum (8/142, 5.6%).

Common complications described were dermatologic complications, including various acute and chronic rashes, nodules and ulcers (59/142, 41.5%), and granulomas (58/142, 40.8%). Other complications included pulmonary complications (51/142, 35.9%) and infections including injection site infections, cellulitis, and abscesses (35/142, 24.6%). The articles reported death in 13.3% (19/142).

Of the 51 articles that identified pulmonary complications, they were described as silicone embolism syndrome, pulmonary embolism, pneumonitis, or diffuse alveolar hemorrhage. Among the substances injected, 92.2% was identified as silicone (47/51), followed by polyacrylamide hydrogel (2/51, 3.9%), hyaluronic acid (1/51, 2%), and castor oil (1/51, 2%).

Regarding the management of complications, antibiotics were documented as being used in 40.1% (57/142) of studies and corticosteroids in 50.7% (72/142). Moreover, 48.3% (60/142) of the studies reported the need for surgery, and 14% (20/142) reported the need for debridement.

Strategies, measures, and lessons to assess illicit liquid silicone injection were mentioned in 20.4% (29/142) of the studies (Table 3). Five main domains were identified:

- Awareness and education (seven statements);
- Medical guidelines (six statements);
- Diagnostic and preventive measures (five statements);
- Public health policies (six statements);
- Regulatory measures (five statements).

**Table 3:** Policies, solutions, and demands identified for the management of illegal silicon injections.

Main domains
**1. Awareness and Education:**
"It is critical to inform the medical community, patients, and non-medical colleagues of the severe, possibly irreparable, consequences of such procedures."
"It is important to consider the need for educational strategies to inform the public of the dangers and damage to one's health involved in going to clandestine clinics that provide aesthetic procedures."
"Our case calls for increased awareness about potential complications..."
"Our case calls for increased awareness about potential complications of silicone injection, as well as increased regulations around unauthorized use by non-medical personnel. "
"Increased public awareness is needed for the prevention of this physically and psychologically debilitating problem."
"Patients should be informed that effects of oil injections."
"Improved surveillance of trans-feminine surgical patients. Preventative measures through screening and imaging of patients with past hormone treatments or injections have the potential to advise the surgical management of transgender individuals and offer excellent esthetic results."
**2. Medical guidelines:**
"Physicians should acknowledge this potential complication of silicone fillers..."
"In patients who admits to a history of silicone or other polymer injection, anesthesia providers should consider obtaining diagnostic imaging such as [magnetic resonance imaging] MRI prior to planned neuraxial anesthesia to assess the condition of the puncture site."
"In any patient with recalcitrant hypercalcemia and a history of known or unsure cosmetic procedures..."
"Silicone embolization syndrome affecting the lungs should always be considered..."
"Physicians and other health care specialists must be vigilant for the misuse of these dermal fillers..."
"Dermatologists should be vigilant about the various complications associated with fillers..."
**3. Diagnostic and preventive measures:**
"In patients who admit to a history of silicone or other polymer injection..."
"Prevention of this practice through public health awareness and education campaigns..."
"Diarrhea, hematuria, and bloody stools can be caused by vaginal wall injection of [polyacrylamide gel] PAAG..."
"A history of cosmetic procedures involving silicone should be considered..."
"BAL and tissue biopsy can help make the diagnosis of silicone pneumonitis."
**4. Public health policies:**
"It is important to consider the need for educational strategies to inform the public..."
"Prevention of this practice through public health awareness and education campaigns in areas of high prevalence is paramount."
"Increased public awareness is needed for the prevention of this physically and psychologically debilitating problem."
"This study highlights the dangers and the inefficiency of clandestine esthetic surgery. There is a need for targeted information campaigns with transgender populations about silicone injections."
"All physicians should be alert to the current cluster of *Mycobaterium abscessus* infections after injections for cosmetic purposes by nonmedical practitioners in New York City."
"From a prevention perspective, alternatives to filler injection such as affordable or insurance-covered cosmetic surgical and dermatological procedures would do much to prevent the incredible complications reported in the review for this study. Such efforts in medical care and prevention are imminently needed as news coverage shows these procedures are rampant and have led to too many deaths for inaction."
**5. Regulatory measures:**
"Physicians should acknowledge this potential complication of silicone fillers..."
"Prevent the use of industrial liquid silicone by unqualified professionals."
"Considering its potential toxicity, the use of silicone by unqualified persons should be prohibited..."
"Soft-tissue filler injections should be administered only by licensed providers..."
"All physicians should be alert to the current cluster of *Mycobaterium abscessus* infections after injections for cosmetic purposes by nonmedical practitioners in New York City."

Source: Elaborated by the authors.

## Discussion

Throughout history, the pursuit of idealized beauty has resulted in body-image dissatisfaction and distortion^12^^–^ 14. In our study, we observed some of the profound impacts that this dissatisfaction, combined with the widespread availability of illicit cosmetic procedures, has had on a global scale.

Body dissatisfaction, combined with the high costs of regulated procedures, often drives patients to take drastic risks, such as turning to unlicensed providers^15^^,^ 16. In our review, half of the articles mentioned that cosmetic injections were performed by an unlicensed provider, and given that 40.1% of studies did not describe the provider’s qualifications. This number could be much higher. Furthermore, only 10.6% of procedures were reported to have occurred in medical facilities, mostly clinics, yet the majority did not specify the location of application. These figures speak to the number of people willing to undergo a medical procedure performed by a person without medical training, outside of a medical facility, to alter their physical appearance.

It is important to highlight that the reasons for patients seeking such procedures include not only perceived beautification, but also gender-affirmation^8^. In our study, 49.9% of patients whose gender was specified were transgender women, exceeding the number of cisgender women and men. Gender affirmative procedures must be understood as an essential aspect of identity, social acceptance, and career advancement to ensure equitable access to gender-affirming care that must be seen as a component of universal health care^17^.

In 2004, the National Coalition for Lesbian, Gay, Bisexual, and Transgender Health recognized the widespread public health concern surrounding silicone injections among male-to-female transgender populations in the United States of America^2^. These informal markets not only emerge as a consequence of economic demand, but also from processes of social exclusion that effectively deny trans individuals access to health care, and in particular transgender care and social services^9^^,^ 17. Ideally, healthcare providers and institutions should support and guide gender transitions. Still, barriers to accessing competent and culturally sensitive health care and a lack of readiness in the medical field and society often lead individuals to take substantial risks^9^^,^ 17.

We hypothesize that the same social and economic barriers to care that steer trans individuals toward illicit cosmetic procedures also contribute to delayed access to care for complications resulting from such procedures. Prior research demonstrates that medically underserved populations, particularly the transgender community, are at higher risk of illicit silicone injection consequences^18^^,^ 19. In our review, we found that the majority of patients whose gender was identified were transgender women, and complications of illicit cosmetic injections included both high-acuity conditions requiring resource-intensive care and chronic sequelae requiring long-term medical and surgical care. Taking this into account, providing comprehensive trans health care is not only essential for valuing human life, but might also be cost-effective.

Another important finding of our review was the lack of provider education and formalized protocols for prompt and effective diagnosis and treatment of patients who suffer complications from illicit cosmetic injections^20^. In some cases, prompt diagnosis is hindered by a lack of disclosure on the patient’s part regarding illicit cosmetic injections they have received. This is likely a result of both social stigma and legal concerns. Regardless, providers should know when to consider illicit cosmetic injection-related complications and should have evidence-based management guidelines to follow.

The injection of liquid silicone for cosmetic purposes has a history of legal and illicit practice worldwide^2^. The most frequent regions registered in our study were the Americas and Asia, which is compatible with existing literature^4^^,^ 6 ^,^ 7. In Puerto Rico, silicone injection is one of the most common procedures used for bodily feminization among trans women and often conducted without clinical supervision or follow-up^17^.

Interestingly none of the studies encountered describing illegal silicone injection cases and the consequences implied were from low-income countries or lower-middle income countries. This may be explained by the systemic paucity of research output from these countries and the decreased availability of standardized data collection and analysis systems^21^.

It is crucial to recognize that the information documented primarily originates from individuals admitted to a hospital and/or who experienced complications, which were then perceived and reported. This approach may lead to underrepresenting vulnerable populations and countries lacking a research culture or data registries^21^^,^ 22. Furthermore, it suggests a potential under-documentation of the actual numbers related to the use of illicit silicone, including uncomplicated cases that were neither seen nor reported.

Our study calls for increased awareness about the potential complications of silicone injection, as well as increased regulations around unauthorized use by non-medical personnel^2^^,^ 23. This study also underscores the importance of detailed medical history and formal guidelines to enhance treatment, diagnosis, and care. Additionally, the absence of formally notifying the cases and the prevalence of inaccurate death certificates underscores the critical need for accurate reporting^22^. Accurate documentation and health data are essential for raising awareness and facilitating effective public health control measures.

From a preventative perspective, alternatives to filler injection such as affordable or insurance-covered cosmetic surgical and dermatological procedures would do much to prevent similar complications to those reported in this review^19^. Existing barriers must be addressed, such as prejudice within health care services, public education, and a lack of knowledge regarding the care for gender minorities^2^.

Despite the comprehensive scope of this review, our study is not exempt from limitations. We only included English, Portuguese, or Spanish citations, possibly excluding relevant research in other languages. Another limitation concerns differences in study design, sample size, and methodology across the included studies, which could affect the generalizability of findings. Although efforts were made to ensure international representation in the selected studies, the overrepresentation of literature from specific geographic regions and high-income and upper-middle-income countries may limit the application of our findings to other populations. Lastly, as a scoping review, we did not assess individual study biases. Future research should consider these limitations to enhance our understanding of the practice and consequences of illicit silicone injection.

## Conclusion

In this scoping review, we comprehensively evaluated illicit silicone injection characteristics, demographics, complications, and possible solutions. Our review synthesized evidence that could guide health care providers and policymakers to better comprehend how to detect and address this potentially dangerous practice and identify and protect the most vulnerable populations.

Our review also highlighted the need to promote awareness and education of at-risk populations regarding the risks of non-regulated procedures while striving to improve this same population’s access to legal, regulated care. There is a need to strengthen and standardize streamlined policies to regulate illicit cosmetic surgical practices worldwide to enhance health care quality.

## Data Availability

Supplementary files are deposited in: https://figshare.com/s/dafff563760592b39c57?file=48314077

## References

[1] Soliman SB (2023). Liquid silicone filler migration following illicit gluteal augmentation. Radiol Case Rep.

[2] Leonardi NR, Compoginis JM, Luce EA (2016). Illicit Cosmetic Silicone Injection: A Recent Reiteration of History. Ann Plast Surg.

[3] Hage JJ, Kanhai RC, Oen AL, van Diest PJ, Karim RB (2001). The devastating outcome of massive subcutaneous injection of highly viscous fluids in male-to-female transsexuals. Plast Reconstr Surg.

[4] Shen Y, Pang Q, Xu J (2021). Long-term complications after liquid silicone injection: A case report and literature review. Chin J Plast Reconst Surg.

[5] Chasan PE (2007). The history of injectable silicone fluids for soft-tissue augmentation. Plast Reconstr Surg.

[6] Bertin C, Abbas R, Andrieu V, Michard F, Rioux C, Descamps V, Yazdanpanah Y, Bouscarat F (2019). Illicit massive silicone injections always induce chronic and definitive silicone blood diffusion with dermatologic complications. Medicine.

[7] Chayangsu O, Wanitphakdeedecha R, Pattanaprichakul P, Hidajat IJ, Evangelista KER, Manuskiatti W. (2020). Legal vs. illegal injectable fillers: The adverse effects comparison study. J Cosmet Dermatol.

[8] Pinto TP, Teixeira F do B, Barros CR dos S, Martins RB, Saggese GSR, Barros DD de, Veras ASM (2017). Silicone líquido industrial para transformar o corpo: prevalência e fatores associados ao seu uso entre travestis e mulheres transexuais em São Paulo, Brasil. Cad Saúde Pública.

[9] Schuylenbergh J, Motmans J, Defreyne J, Somers A, T’Sjoen G (2019). Sexual health, transition-related risk behavior and need for health care among transgender sex workers. Int J Transgend.

[10] Tricco AC, Lillie E, Zarin W, O’Brien KK, Colquhoun H, Levac D, Moher D, Peters MDJ, Horsley t, Weeks L, Hempel S, Akl EA, Chang C, McGowan J, Stewart L, Hartling L, Aldcroft A, Wilson MG, Garritty C, Lewin S, Godfrey CM, Macdonald MT, Langlois EV, Soares-Weiser K, Moriarty J, Clifford T, Tunçalp O, Straus SE (2018). PRISMA Extension for Scoping Reviews (PRISMA-ScR): Checklist and Explanation. Ann Intern Med.

[11] Covidence (2020). Covidence: Better systematic review management.

[12] Madan S, Basu S, Ng S, Ching Lim EA (2018). Impact of Culture on the Pursuit of Beauty: Evidence from Five Countries. J Int Mark.

[13] Rajanala S, Maymone MBC, Vashi NA (2020). Evolving beauty-Creating and transforming inequalities. J Cosmet Dermatol.

[14] Fakih N, Bertossi D, Vent J (2022). The Overfilled Face. Facial Plast Surg.

[15] Sarwer DB (2021). Returning for Aesthetic Procedures: Compliance or Compulsion?. Aesthet Surg J.

[16] Mayer JE, Goldberg DJ (2015). Injuries Attributable to Cosmetic Procedures Performed by Unlicensed Individuals in the United States. J Clin Aesthet Dermatol.

[17] Padilla MB, Rodríguez-Madera S, Ramos Pibernus AG, Varas-Díaz N, Neilands TB (2018). The social context of hormone and silicone injection among Puerto Rican transwomen. Cult Health Sex.

[18] Sergi FD, Wilson EC (2021). Filler Use Among Trans Women: Correlates of Feminizing Subcutaneous Injections and Their Health Consequences. Transgend Health.

[19] Wilson E, Rapues J, Jin H, Raymond HF (2014). The Use and Correlates of Illicit Silicone or “Fillers” in a Population‐Based Sample of Transwomen, San Francisco, 2013. J Sex Med.

[20] Zheng C, Quentzel J, Brust JCM (2019). Complications of Silicone Cosmetic Procedures Among Medical Tourists from the Bronx, New York: A Retrospective Analysis. J Clin Aesthet Dermatol.

[21] Mughal NA, Hussain MH, Ahmed KS, Waheed MT, Munir MM, Diehl TM, Zafar SN (2023). Barriers to Surgical Outcomes Research in Low- and Middle-Income Countries: A Scoping Review. J Surg Res.

[22] Di Santis ÉP, Yarak S, Martins MR, Hirata SH (2022). Compulsory notification of injuries in aesthetic procedures. Impact on patient safety. An Bras Dermatol.

[23] Schenone GE, Riera DN, Fontbona M, Triana L (2023). US FDA Safety Communication on Illegal use of Injectable Silicone for Body Contouring and Associated Health Risks. Aesthetic Plast Surg.

